# Cyanobacteria *Scytonema javanicum* and *Scytonema ocellatum* Lipopolysaccharides Elicit Release of Superoxide Anion, Matrix-Metalloproteinase-9, Cytokines and Chemokines by Rat Microglia In Vitro

**DOI:** 10.3390/toxins10040130

**Published:** 2018-03-21

**Authors:** Lucas C. Klemm, Evan Czerwonka, Mary L. Hall, Philip G. Williams, Alejandro M. S. Mayer

**Affiliations:** 1 Biomedical Sciences Program, College of Health Sciences, Midwestern University, Downers Grove, IL 60515, USA; lklemm@wisc.edu; 2 Department of Pharmacology, Chicago College of Osteopathic Medicine, Midwestern University, Downers Grove, IL 60515, USA; eczerwonka79@midwestern.edu (E.C.); mhallx@midwestern.edu (M.L.H.); 3 Department of Chemistry, University of Hawaii at Manoa, Honolulu, HI 96882, USA; philipwi@hawaii.edu

**Keywords:** microglia, cyanobacterium, *Scytonema*, lipopolysaccharide, cytokine, chemokine, superoxide, MMP-9, rat

## Abstract

Cosmopolitan Gram-negative cyanobacteria may affect human and animal health by contaminating terrestrial, marine and freshwater environments with toxins, such as lipopolysaccharide (LPS). The cyanobacterial genus *Scytonema* (*S*) produces several toxins, but to our knowledge the bioactivity of genus *Scytonema* LPS has not been investigated. We recently reported that cyanobacterium *Oscillatoria* sp. LPS elicited classical and alternative activation of rat microglia in vitro. Thus, we hypothesized that treatment of brain microglia in vitro with either cyanobacteria *S. javanicum* or *S. ocellatum* LPS might stimulate classical and alternative activation with concomitant release of superoxide anion (O_2_^−^), matrix metalloproteinase-9 (MMP-9), cytokines and chemokines. Microglia were isolated from neonatal rats and treated in vitro with either *S. javanicum* LPS, *S. ocellatum* LPS, or *E. coli* LPS (positive control), in a concentration-dependent manner, for 18 h at 35.9 °C. We observed that treatment of microglia with either *E. coli* LPS, *S. javanicum* or *S. ocellatum* LPS generated statistically significant and concentration-dependent O_2_^−^, MMP-9 and pro-inflammatory cytokines IL-6 and TNF-α, pro-inflammatory chemokines MIP-2/CXCL-2, CINC-1/CXCL-1 and MIP-1α/CCL3, and the anti-inflammatory cytokine IL-10. Thus, our results provide experimental support for our working hypothesis because both *S. javanicum* and *S. ocellatum* LPS elicited classical and alternative activation of microglia and concomitant release of O_2_^−^, MMP-9, cytokines and chemokines in a concentration-dependent manner in vitro. To our knowledge this is the first report on the toxicity of cyanobacteria *S. javanicum* and *S. ocellatum* LPS to microglia, an immune cell type involved in neuroinflammation and neurotoxicity in the central nervous system.

## 1. Introduction

Cyanobacteria are photoautotrophic Gram-negative bacteria that are found in a wide range of terrestrial, marine and freshwater environments [1]. Overgrowth of cyanobacteria can result in blooms which may include cyclic hepatotoxic peptides, neurotoxic alkaloids and LPS [2], which can affect human health [3,4] through various routes, including drinking, skin and respiratory exposure, or via the circulatory system [5,6].

The cyanobacterial genus *Scytonema* has been reported to produce several types of toxins: tolytoxin, a member of the polyketide-derived macrolides scytophycins, that displayed cytotoxic and antifungal activity [7,8,9]; scytovirin, a novel anti-HIV protein [10]; an antimicrobial sesterpene, scytoscalarol [11], the cyclic peptides scytonemides A and B, with 20S proteasome inhibitory activity [12], and more recently, the alkaloid saxitoxins, fast-acting neurotoxins that block sodium channels [13,14]. To our knowledge, the bioactivity of cyanobacteria *S. javanicum* and *S. ocellatum* LPS has not been investigated.

One body system that may be affected by cyanobacterial LPS is the central nervous system (CNS), which has long been considered an immunologically privileged site [15], although the peripheral immune system may communicate with microglia, the macrophages of the brain immune system, via neural and humoral routes [16]. Microglia are dedicated macrophages of the CNS which originate in the yolk-sac, then migrate from the blood system to the brain during early development, and play an important role in brain homeostasis [17].

Two microglia activation states, termed classical and alternative, appear to enable microglia to react to stimuli and restore tissue homeostasis [18]. Classically activated or M1 microglia, that may be induced by LPS [19], are characterized by production of pro-inflammatory chemokines and cytokines, such as tumor necrosis factor (TNF-α), interleukin-6 (IL-6), interleukin-1β (IL-1β), and interferon-γ [20], and are involved in neuroinflammation [21]. Alternatively activated, or M2 microglia, down-regulate the inflammatory response and generate anti-inflammatory cytokines such as IL-4, IL-10, IL-13, and transforming growth factor-β [21].

The structure of LPS, an outer membrane component of Gram-negative bacteria [22], consists of an O-antigen, a core, and lipid A [23]. Lipid A is composed of units of D-glucosamine dimers and fatty acid chains, anchors LPS to the membrane, and is responsible for the toxicity of LPS [23]. Lipid A differences between Gram-negative proteobacteria and cyanobacteria [24,25] appear to affect its functionality [26,27], and have been proposed to result in diminished toxicity [25,28].

The purpose of this study was to test the hypothesis that in vitro treatment of primary neonatal rat microglia with *S. javanicum* or *S. ocellatum* LPS might trigger classical (or M1-type) and/or alternative (or M2-type) microglia activation and the concomitant release of the pro-inflammatory mediators O_2_^−^, thromboxane B_2_ (TXB_2_) and MMP-9, as well as cytokines TNF-α and IL-6, chemokines MIP-1α/CCL3, MIP-2/CXCL-2 and CINC-1/CXCL-1, and the anti-inflammatory cytokine IL-10. Our results support our working hypothesis because both *S. javanicum* and *S. ocellatum* LPS activated both classical (or M1-type) and alternative (or M2-type) phenotypes of rat microglia in vitro, in a concentration-dependent manner. Thus, our investigation, the first to report on the immunotoxicity of cyanobacteria *S. javanicum* and *S. ocellatum* LPS to brain microglia, extends current knowledge of the toxicology of the cyanobacterial genus *S. cytonema*.

## 2. Results

### 2.1. Effect of S. javanicum and S. ocellatum LPS on Neonatal Rat Brain Microglia O_2_^−^ Generation

**Figure 1:**
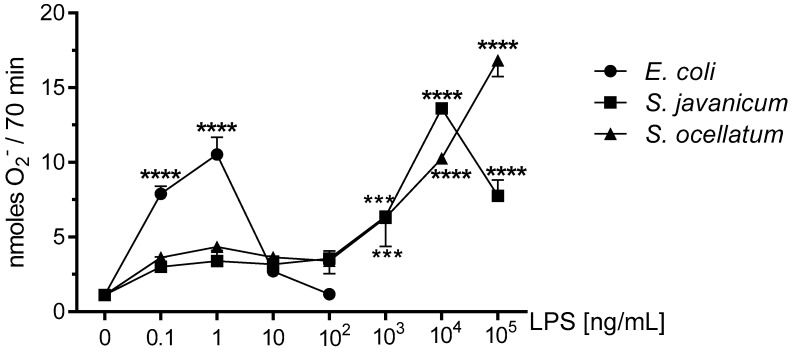
The effect of *E. coli*, *S. javanicum* and *S. ocellatum* LPS on neonatal rat microglia O_2_^−^ release. Neonatal rat microglia (1.8–2.0 × 10^5^ cells/well) were treated with *E. coli* LPS (0.1–100 ng/mL), *S. javanicum* or *S. ocellatum* LPS (0.1–10^5^ ng/mL) for 18-h in vitro and then stimulated with PMA (1 μM) for 70 min. O_2_^−^ was determined as described in the Materials and Methods section. Data expressed as nanomoles O_2_^−^ is the mean ± SEM from three independent experiments (n), each experiment with triplicate determinations. *** *p* < 0.001, **** *p* < 0.0001 LPS versus untreated control (0).

Reactive oxygen species generated by microglia can cause neuronal injury via oxidative stress and have been implicated in neurodegenerative diseases [19,21,29]. Previous work from our laboratory has reported that rat microglia treated in vitro with either *E. coli* LPS [19], cyanobacteria *Microcystis aeruginosa* or *Oscillatoria* sp. LPS release O_2_^−^ in vitro [3,4]. As shown in Figure 1, PMA-stimulated O_2_^−^ release was observed when neonatal rat microglia were treated with either *E. coli*, *S. javanicum* or *S. ocellatum* LPS for 18 h. Maximal O_2_^−^ release was observed at 1 × 10 ^4^ ng/mL *S. javanicum* LPS and 1 × 10^5^ ng/mL *S. ocellatum* LPS. In contrast, *E. coli* LPS, the positive control, showed maximal O_2_^−^ release at 1 ng/mL as previously reported [3]. Thus, *S. javanicum* and *S. ocellatum* LPS appeared to be 10,000- and 100,000-fold, respectively, less potent than *E. coli* LPS in stimulating statistically significant O_2_^−^ production from neonatal rat microglia in vitro.

### 2.2. Effect of S. javanicum and S. ocellatum LPS on Neonatal Rat Brain Microglia LDH Generation

To determine whether the decrease in O_2_^−^ production shown in Figure 1 resulted from concentration-dependent toxicity from *E. coli* or *Scytonema* LPS to microglia during the 18 h incubation, LDH release was determined in culture supernatants [19]. LDH release has been used extensively as a marker for cellular toxicity, as is described in the Materials and Methods [3,4].

**Figure 2:**
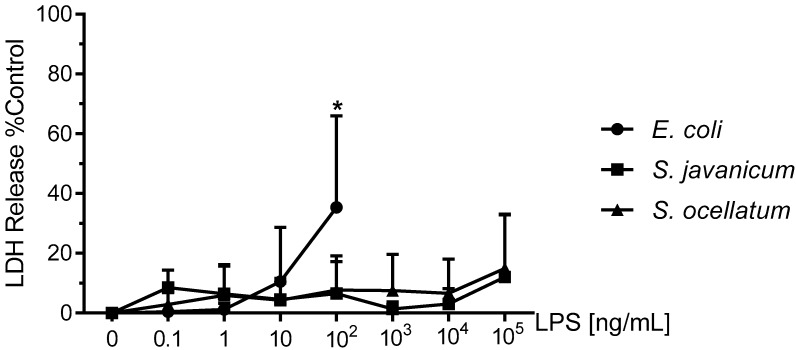
The effect of *E. coli*, *S. javanicum* and *S. ocellatum* LPS on neonatal rat microglia LDH release. Neonatal rat microglia (1.8–2.0 × 10^5^ cells/well) were treated with *E. coli* LPS (0.1–100 ng/mL), *S. javanicum* or *S. ocellatum* LPS (0.1–10^5^ ng/mL) for 18-h in vitro. LDH release was determined as described in the Materials and Methods section. Data expressed as % of 0.1% Triton X-100-treated microglia LDH release, is the mean ± SEM from three independent experiments (n), each experiment with triplicate determinations. * *p* < 0.05 LPS versus untreated control (0).

As shown in Figure 2, LDH release was low for all concentrations of both *S. javanicum* and *S. ocellatum* LPS we investigated. In *S. javanicum* and *S. ocellatum*-LPS treated microglia, a non-statistically significant release of LDH was observed at 100,000 ng/mL LPS, reaching 12.1 ± 12.1% and 14.9 ± 10.5% of control, respectively. In contrast, in *E. coli* LPS-stimulated microglia, a statistically significant LDH release of 35.3 ± 17.7% of control was observed at 100 ng/mL, as previously reported [3]. Thus, the LDH data suggest that both *S. javanicum* and *S. ocellatum* LPS elicited classical and alternative activation of microglia and concomitant release of O_2_^−^, MMP-9 and cytokines and chemokines in a concentration-dependent manner while not affecting the microglia cell membrane in vitro at the concentrations tested in these experiments.

### 2.3. Effect of S. javanicum and S. ocellatum LPS on Neonatal Rat Brain Microglia Proinflammatory TXB_2_ Generation

Eicosanoids, such as TXB_2_, have been implicated in neurodegenerative disease by contributing to neuroinflammation [30]. We have reported that cyanobacteria *Microcystis aeruginosa* and *Oscillatoria* sp. LPS stimulated rat microglia to release TXB_2_ in vitro [3,4,19]. As shown in Supplementary Table S1, both *S. javanicum*, and *S. ocellatum* LPS-treated microglia showed a non-statistically significant TXB_2_ release as compared to untreated microglia.

### 2.4. Effect of S. javanicum and S. ocellatum LPS on Neonatal Rat Brain Microglia Pro-Inflammatory MMP-9 Generation

**Figure 3:**
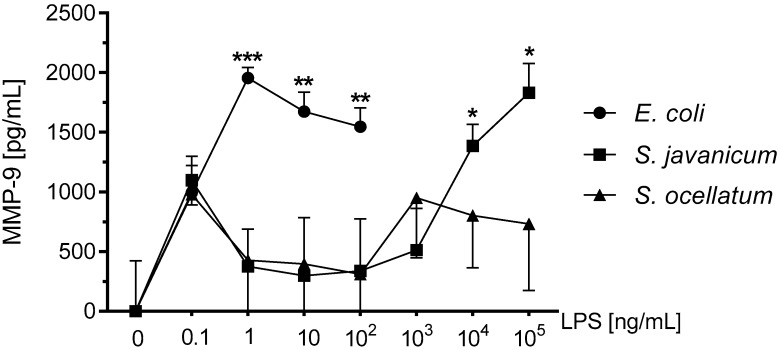
The effect of *E. coli, S. javanicum* and *S. ocellatum* LPS on neonatal rat microglia MMP-9 release. Neonatal rat microglia (1.8–2.0 × 10^5^ cells/well) were treated with *E. coli* LPS (0.1–100 ng/mL), *S. javanicum* or *S. ocellatum* LPS (0.1–10^5^ ng/mL) for 18-h in vitro. MMP-9 release was determined as described in the Materials and Methods section. Basal release (0 ng/mL LPS) of 3070.5 pg/mL MMP-9 was substracted from all data. Data expressed as pg/mL is the mean ± SEM from three independent experiments (n), each experiment with triplicate determinations. * *p* < 0.05, ** *p* < 0.01, *** *p* < 0.001 LPS versus untreated control (0).

MMP-9 and other matrix metalloproteinases produced during neuroinflammation may affect the blood brain barrier causing disruption and resulting neuropathology [31]. Our laboratory has previously reported that rat microglia release MMP-9 upon stimulation with cyanobacteria *Microcystis aeruginosa* and/or *Oscillatoria* sp. LPS [4,19]. MMP-9 release in supernatants was measured via ELISA. As shown in Figure 3, *E. coli* LPS-treated microglia released statistically significant levels of MMP-9 from 1–100 ng/mL LPS. *S. javanicum* LPS-treated microglia also released statistically significant levels of MMP-9 but at 10,000–100,000 ng/mL LPS. In contrast, *S. ocellatum* LPS did not induce statistically significant release of MMP-9 from treated microglia. Thus, *S. javanicum* LPS appeared to be 10,000-fold, less potent than *E. coli* LPS in stimulating statistically significant MMP-9 production from neonatal rat microglia in vitro.

### 2.5. Effect of S. javanicum and S. ocellatum LPS on Neonatal Rat Brain Microglia Proinflammatory Cytokine Release: TNF-α and IL-6 

**Figure 4:**
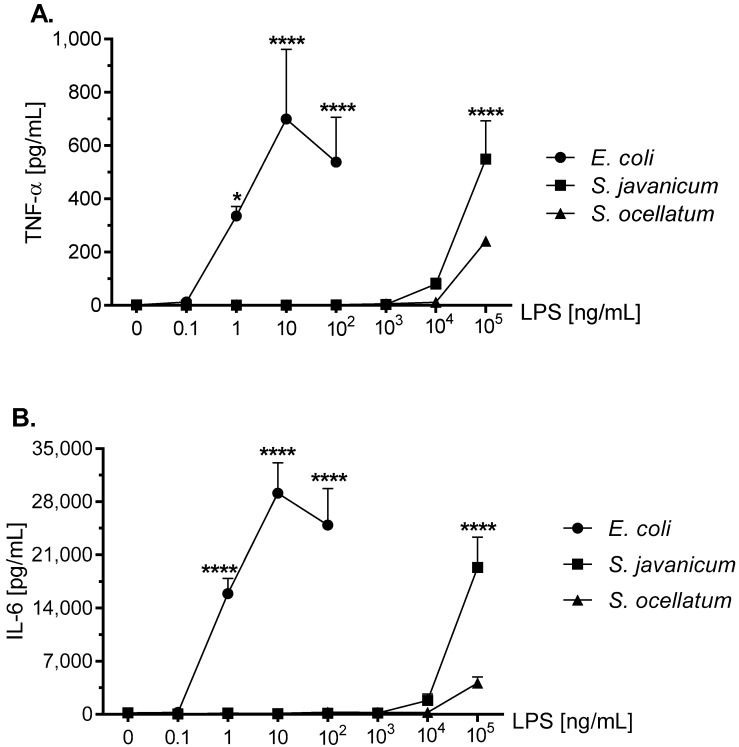
The effect of *E. coli*, *S. javanicum* and *S. ocellatum* LPS on neonatal rat microglia TNF-α (panel **A**) and IL-6 (panel **B**) release. Neonatal rat microglia (1.8–2.0 × 10^5^ cells/well) were treated with *E. coli* LPS (0.1–100 ng/mL), *S. javanicum* or *S. ocellatum* LPS (0.1–10^5^ ng/mL) for 18-h in vitro. TNF-α and IL-6 release were determined as described in the Materials and Methods section. Data expressed as pg/mL are the mean ± SEM from three independent experiments (n), each experiment with triplicate determinations. * *p* < 0.05, **** *p* < 0.0001 LPS versus untreated control (0).

TNF-α is a pro-inflammatory cytokine that appears to play a role in neurodegenerative diseases [21]. Release of TNF-α from LPS-stimulated microglia has been demonstrated in primary rat microglia [32,33]. As shown in Figure 4, panel A, microglia stimulated with *E. coli* LPS for 18 h in vitro showed a statistically significant peak TNF-α release at 10 ng/mL LPS (699.4 ± 262.1 pg/mL). Similarly, *S. javanicum* LPS-stimulated microglia released statistically significant TNF-α at 1 × 10^5^ ng/mL (549.2 ± 144.9 pg/mL; *p* < 0.0001), while in contrast *S. ocellatum* LPS-stimulated TNF-α release was non-statistically significant (240.8 ± 13.3 pg/mL).

IL-6 is a pro-inflammatory cytokine involved in cellular survival, stress responses, and may also contribute to neuroinflammation [34]. LPS-stimulated rat microglia may release IL-6 [32,33,35]. As shown in Figure 4, panel B, the concentration-dependent release of IL-6 was similar to TNF-α (Figure 4, panel A) in LPS-treated rat microglia, although differed in the total magnitude (pg/mL) generated. Thus *E. coli* LPS-stimulated microglia released peak IL-6 at 10 ng/mL LPS (29,117.7 ± 3998.3 pg/mL IL-6; *p* < 0.0001) while in contrast, *S. javanicum* LPS-treated microglia IL-6 generation peaked at 1×10^5^ ng/mL LPS (19,340 ± 3973.0 pg/mL IL-6; *p* < 0.0001). Furthermore, and similar to TNF-α release, *S. ocellatum* LPS-triggered IL-6 generation at 1 × 10^5^ ng/mL LPS, was non-statistically significant (4118.9 ± 797.2 pg/mL). Thus, like O_2_^−^ and MMP-9 release, cyanobacteria *S. javanicum* and *S. ocellatum* LPS appeared to be approximately 10,000 fold less potent than *E. coli* LPS in stimulating rat microglia to release classical activation cytokines TNF-α and IL-6 in vitro.

### 2.6. Effect of S. javanicum and S. ocellatum LPS on Neonatal Rat Brain Microglia Proinflammatory Chemokine Release: MIP-1α/CCL3, CINC-1/CXCL-1, and MIP-2/CXCL-2

**Figure 5:**
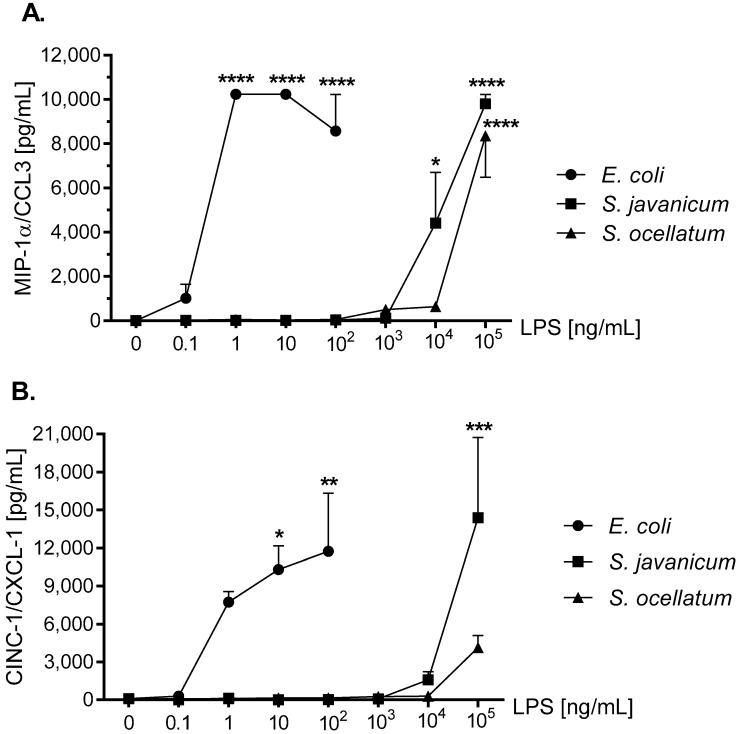

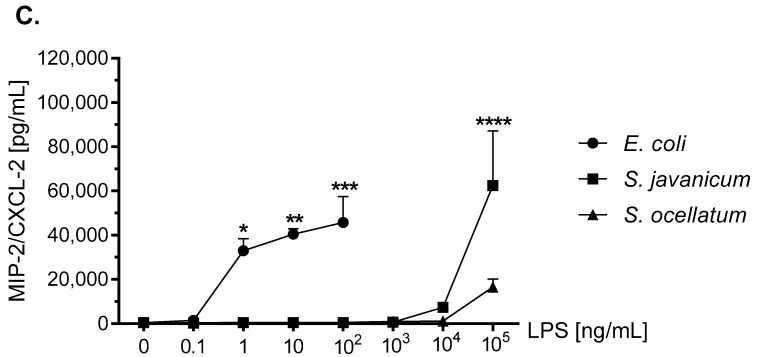
The effect of *E. coli*, *S. javanicum* and *S. ocellatum* LPS on neonatal rat microglia MIP-1α/CCL3 (panel **A**), CINC-1/CXCL-1 (panel **B**), and MIP-2/CXCL-2 (panel **C**) release. Neonatal rat microglia (1.8–2.0 × 10^5^ cells/well) were treated with *E. coli* LPS (0.1–100 ng/mL), *S. javanicum* or *S. ocellatum* LPS (0.1–10^5^ ng/mL) for 18-h in vitro. MIP-1α/CCL3, CINC-1/CXCL-1, and MIP-2/CXCL-2 release was determined as described in the Materials and Methods section. Data expressed as pg/mL is the mean ± SEM from two or three independent experiments (n), each experiment with triplicate determinations. * *p* < 0.05, ** *p* < 0.01, *** *p* < 0.001, **** *p* < 0.0001 LPS versus untreated control (0).

MIP-1α/CCL3, a neuroinflammation biomarker, has been shown to recruit granulocytes to damaged brain regions [36]. MIP-1α/CCL3 has been reported to be generated by LPS-stimulated mouse [37], human [38], and rat microglia [39]. As shown in Figure 5, panel A, after an 18 h in vitro incubation with either *E. coli*, *S. javanicum* or *S. ocellatum* LPS, rat microglia generated MIP-1α/CCL3. Thus at 1 and 10 ng/mL *E. coli* LPS-treated rat microglia released 10,235 ± 6.5 pg/mL MIP-1α/CCL3, *p* < 0.0001. In contrast, 100,000 ng/mL *S. javanicum* and *S. ocellatum* LPS-treated microglia generated 9806.8 ± 422.3 and 8357.2 ± 1871.8 pg/mL MIP-1α/CCL3, *p* < 0.0001, respectively.

CINC-1/CXCL-1 is involved in the chemotaxis and activation of neutrophils [40]. CINC-1/CXCL-1 release from LPS-stimulated microglia has been observed in rat [41,42] and mouse [37,43]. As shown in Figure 5, panel B, 100 ng/mL *E. coli* LPS-treated rat microglia showed maximal CINC-1/CXCL-1 release of 11,742.1 ± 4593.4 pg/mL, *p* < 0.01. Furthermore, 1 × 10^5^ ng/mL *S. javanicum* LPS though less potent, turned out to be more efficacious with a maximal release of 14,387.9 ± 6343 pg/mL CINC-1/CXCL-1, *p* < 0.001. Surprisingly, 1 × 10^5^ ng/mL *S. ocellatum* LPS-treated microglia showed a non-statistically significant CINC-1/CXCL-1 release of 4132.6 ± 947.8 pg/mL.

MIP-2/CXCL-2 is another neutrophil chemoattractant and activator [44]. LPS-stimulated mouse [45,46] and rat microglia release MIP-2/CXCL-2 [35,42]. As seen in Figure 5, panel C, 100 ng/mL *E. coli* LPS induced peak release of 45,710.2 ± 11,774.2 pg/mL MIP-2/CXCL-2, *p* < 0.001, while 1 × 10^5^ ng/mL *S. javanicum* LPS-treated microglia showed statistically significant release of 62,423.9 ± 24,688.2 pg/mL MIP-2/CXCL-2, *p* < 0.0001. In contrast, and similar to what was observed with CINC-1/CXCL-1 generation, at 1 × 10^5^ ng/mL *S. ocellatum* LPS-treated microglia generated non-statistically significant MIP-2/CXCL-2 (16,550.6 ± 3550.9 pg/mL).

Thus, similar to cytokines TNF-α and IL-6, cyanobacteria *S. javanicum* and *S. ocellatum* LPS appeared to be approximately 10,000 fold less potent than *E. coli* LPS in stimulating rat microglia to release both the pro-inflammatory CXCL chemokine MIP-2/CXCL2, and the pro-inflamatory CCL chemokines CINC-1/CXCL-1 and MIP-2/CXCL-2 in vitro. 

### 2.7. Effect of S. javanicum and S. ocellatum LPS on Neonatal Rat Brain Microglia Anti-Inflammatory Cytokine Release: IL-10

**Figure 6:**
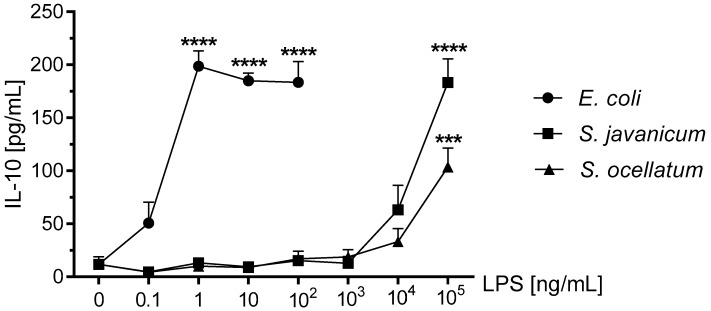
The effect of *E. coli, S. javanicum* and *S. ocellatum* LPS on neonatal rat microglia IL-10 release. Neonatal rat microglia (1.8–2.0 × 10^5^ cells/well) were treated with *E. coli* LPS (0.1–100 ng/mL), *S. javanicum* or *S. ocellatum* LPS (0.1–10^5^ ng/mL) for 18-h in vitro. IL-10 release was determined as described in the Materials and Methods section. Data expressed as pg/mL is the mean ± SEM from three independent experiments (n), each experiment with triplicate determinations. *** *p* < 0.001, **** *p* < 0.0001 LPS versus untreated control (0).

The anti-inflammatory cytokine IL-10 has immunosuppressive functions [47] and has been reported to be released by LPS-treated mouse [47], rat [48], and human microglia [49]. As shown in Figure 6, treatment of microglia with *E. coli* LPS resulted in a maximal release of 198.7 ± 14.4 pg/mL IL-10 at 1 ng/mL LPS (*p* < 0.001). Furthermore, both *S. javanicum* and *S. ocellatum*-LPS treated microglia showed statistically significant release of IL-10 at 1 × 10^5^ ng/mL: 183.2 ± 22.2 (*p* < 0.0001) and 103.6 ± 7.3 (*p* < 0.001) pg/mL, respectively.

## 3. Discussion

Microglia activated by stimuli such as infections [50] and neurodegenerative diseases [51] display either the pro-inflammatory M1 or the anti-inflammatory M2 phenotypes that participate in the initiation and resolution of inflammation [43]. One activator of microglia is LPS which activates microglia via its lipid A moiety resulting in the concomitant generation of inflammatory mediators including matrix metalloproteases, arachidonic acid metabolites, cytokines, chemokines, and free radicals both in vivo and in vitro [19].

Our working hypothesis was that cyanobacteria *S. javanicum* and *S. ocellatum* LPS would induce an M1 or classical activation phenotype in primary neonatal rat microglia in vitro and O_2_^−^ release. In fact, both *S. javanicum* and *S. ocellatum* LPS stimulated microglia released statistically significant O_2_^−^ in a concentration-dependent manner similar to *E. coli* LPS, which was used as a positive control. Our present observations are consistent with published observations on O_2_^−^ release by cyanobacteria *Microcystis aeruginosa* and *Oscillatoria* sp. LPS-treated primary rat microglia in vitro [3,4]. While cyanobacterial LPS from either *M. aeruginosa*, *Oscillatoria* sp., or *S. javanicum* showed similar O_2_^−^ release, *S. ocellatum* LPS caused microglia to generate slightly higher concentrations of O_2_^−^. Furthermore, peak O_2_^−^ release was observed at 1000 ng/mL *M. aeruginosa* and *Oscillatoria* sp. LPS [3,4], while in the current study, maximal O_2_^-^ release required 10,000 ng/mL *S. javanicum* LPS and 100,000 ng/mL *S. ocellatum* LPS. The nature of the observed range of potencies among these cyanobacterial LPS and O_2_^−^ release in vitro remains to be investigated in future studies.

In addition to O_2_^−^, *S. javanicum* and *S. ocellatum* LPS-treated microglia generated pro-inflammatory cytokines and chemokines in a concentration-dependent manner: MIP-2/CXCL-2 > IL-6 > CINC-1/CXCL-1 > MIP-1α/CCL3 > TNF-α. Although *S. javanicum* LPS was less potent than *E. coli* LPS in inducing the M1 phenotype, and less efficacious in stimulating release of four cytokines and chemokines, the release of CINC-1/CXCL-1 was enhanced compared to *E. coli* LPS. In contrast, *S. ocellatum* LPS, with the sole exception of MIP-1α/CCL3, was both less potent and less efficacious in activating an M1 microglia phenotype with concomitant release of MIP-2/CXCL-2, IL-6, CINC-1/CXCL-1, and TNF-α.

Two recent studies characterizing microglial activation by cyanobacteria *M. aeruginosa* and *Oscillatoria* sp. LPS [3,4] allow for an interesting comparison of cyanobacterial LPS efficacy and potency in the concomitant generation of pro-inflammatory cytokines and chemokines. Of the four cyanobacterial LPS our laboratory has studied so far, *M. aeruginosa* appears to be the most efficacious in stimulating secretion of MIP-2/CXCL-2, IL-6, MIP-1α/CCL3, and TNF-α, while *S. ocellatum* LPS was the least efficacious. As compared to *Oscillatoria* sp. LPS [3], *S. javanicum* LPS appeared to be more efficacious in stimulating secretion of MIP-2/CXCL-2, IL-6, and CINC-1/CXCL-1 from rat microglia, but resulted in similar concentrations of MIP-1α/CCL3 and TNF-α. Thus, our study provides experimental support for our working hypothesis, namely that cyanobacteria *S. javanicum* and *S. ocellatum* LPS (0.1–100,000 ng/mL) activated an M1 or classical activation phenotype in primary rat microglia, with no significant toxicity to microglia in vitro.

The second component of our working hypothesis was to investigate whether *S. javanicum* and *S. ocellatum* LPS-treated rat microglia activated a M2 or alternative activation phenotype with concomitant release of the anti-inflammatory mediator IL-10. The M2 microglia phenotype and anti-inflammatory mediators have been associated with tissue repair processes [52]. Both *S. javanicum* and *S. ocellatum* LPS-treated rat microglia demonstrated statistically significant concentration-dependent release of the anti-inflammatory cytokine IL-10. Although both *S. javanicum* and *S. ocellatum* LPS were less potent than *E. coli* LPS in stimulating release of IL-10, *S. javanicum* LPS had similar efficacy as *E. coli* LPS. Thus, our present results complement our recently published study on the effects of cyanobacterium *Oscillatoria* sp. LPS on alternative activation of rat microglia and concomitant IL-10 release [3]. In terms of potency, both *Scytonema* species LPS were 10-fold less potent as they did not stimulate maximal IL-10 release until 100,000 ng/mL LPS, whereas *Oscillatoria* sp. LPS resulted in peak IL-10 release at 10,000 ng/mL LPS [3]. We currently hypothesize that the observed differences in potency and efficacy amongst the cyanobacterial LPS could be due to differing lipid A structures [25]. The structures of most cyanobacterial LPS is currently unknown, so determination of LPS structure appears necessary for further characterization of their in vitro and in vivo effects on microglial activation states [25,26].

Taken together, our data lend support to our working hypothesis by demonstrating that in vitro treatment of primary neonatal rat microglia with cyanobacteria *S. javanicum* and *S. ocellatum* LPS will result in both classical or M1 and alternative or M2 activation in a concentration-dependent manner. As our current study was conducted in vitro, and because it has been reported that *E. coli* and *Salmonella tiphyimurium* LPS activate microglia upon systemic administration [53,54,55], future studies are required to determine whether systemic cyanobacterial *S. javanicum* and *S. ocellatum* LPS will activate microglia in the CNS, as well as concomitant pro-inflammatory and anti-inflammatory mediator release.

## 4. Conclusions

In conclusion, the present investigation on the toxicology of both *S. javanicum* and *S. ocellatum* LPS to microglia in vitro extends our previous studies with cyanobacteria *Microcystis aeruginosa* and *Oscillatoria* sp. LPS, and contributes to our understanding of the potential toxicity of cyanobacterial LPS in general, and the genus *Scytonema* in particular, to the brain immune system.

## 5. Materials and Methods

### 5.1. Chemicals

*Escherichia coli* LPS (*Ec*) (026:B6) was purchased from Difco Laboratories (Detroit, MI, USA). Cyanobacteria *S. javanicum* (167 EU/ng) and *S. ocellatum* (77 EU/ng) LPS were prepared by hot phenol/water extraction [56] by Dr. Philip Williams, University of Hawaii at Manoa (Honolulu, HI, USA) from UHM’s strains GB-9-9 and HX-22-2, respectively; endotoxins were assessed using the Genscript ToxinSensor Chromogenic LAL Endotoxin Assay (Piscataway, NJ, USA) that detects the amount of lipid A present. The inherent variability of the LAL assay is 50–200% and can be effected by variations in the structure of lipid A, the degree of aggregation of the LPS sample, and inherent variability in the LAL reagent (lysate of the horseshoe crab) [57,58]; Dulbecco’s modified Eagle medium (DMEM) with high glucose (4.5 mg/L), Hanks’ balanced salt solution (HBSS), penicillin (P), streptomycin (S), and trypsin (0.25%)-EDTA (1 mM) were from GIBCO Laboratories (Life Technologies Inc., Grand Island, NY, USA); heat-inactivated fetal bovine serum certified (FBS) was from Hyclone (Logan, UT, USA). Ferricytochrome c type III (from horse heart) (FCC), superoxide dismutase (from bovine liver) (SOD), phorbol 12-myristate 13-acetate (PMA) and dimethyl sulfoxide (DMSO) were from Sigma Chemical Co. (St. Louis, MO, USA). PMA was maintained at −20 °C as a 10 mM stock solution in DMSO.

### 5.2. LPS Contamination

Glassware and metal spatulas were baked for 4 h at 210 °C to inactivate LPS [59]. Sterile and LPS-free 225-cm^2^ vented cell culture flasks were from BD Biosciences (San Jose, CA, USA); 24-well flat-bottom culture clusters were from Costar^®^ (Corning Inc., Corning, NY, USA); disposable serological pipettes were from Greiner Bio-One (Monroe, NC, USA). Sterile and pyrogen-free Eppendorf Biopur pipette tips were from Brinkmann Instruments, Inc. (Westbury, NY, USA).

### 5.3. Isolation of Primary Rat Neonatal Microglia

Adherence to the National Institutes of Health guidelines on the use of experimental animals and protocols approved by Midwestern University’s Research and Animal Care Committee were followed in all experiments (Midwestern University Protocol File # 941.1 titled “ Neuroinflammation, microglia and marine natural products”was approved on 5 January 2015). The cerebral cortices of 1–2 day-old Sprague-Dawley rats (Charles Rivers, Hartford, CT, USA), were surgically removed, placed in cold DMEM containing 120 U/mL P and 12 μg/mL S, the meninges removed, and brain tissue minced and dissociated with trypsin-EDTA at 35.9 °C for 3–5 min. The mixed glial cell suspension was plated in 225-cm^2^ vented cell culture flasks with DMEM medium supplemented with 10% FBS containing 120 U/mL P and 12 μg/mL S and grown in a humidified 5% CO_2_ incubator at 35.9 °C for 12–14 days. Upon confluence of the astrocyte layer (day 14) and every week thereafter, microglia were detached using an orbital shaker (150 rpm, 0.5 h, 35.9 °C, 5% CO_2_), centrifuged (400× *g*, 25 min, 4 °C), and cell number and viability assessed by trypan blue dye exclusion. Rat neonatal microglia (2 × 10^5^ cells/well) averaging greater than 95% viability were plated in non-pyrogenic polystyrene 24-well flat-bottom culture clusters with DMEM supplemented with 10% FBS containing 120 U/mL P and 12 μg/mL S, and then transferred to a humidified incubator at 35.9 °C and 5% CO_2_, 24 h prior to the experiments. The purity of the isolated rat brain neonatal microglia was routinely determined using a mouse monoclonal anti-rat CD11b antibody (Cat # MCA275R, AbD SeroTec, Raleigh, NC, USA), and was greater than 98%.

### 5.4. Activation of Microglia with LPS (Experimental Protocol)

To determine the effect of *S. javanicum* and *S. ocellatum* LPS on neonatal rat microglia classical and alternative activation and concomitant mediator release (O_2_^−^, thromboxane B_2_, cytokines, and chemokines), 1.8–2.0 × 10^5^ neonatal microglia in DMEM + 10% FBS + 0.1% P + S were plated into each well of a 24-well flat-bottom culture cluster, and then stimulated with either 0.1–100,000 ng/mL cyanobacterium *S. javanicum* LPS, *S. ocellatum* LPS, or *E. coli* LPS (0.1–100 ng/mL) used as a positive control. Time-of-stimulation with *S. javanicum*, *S. ocellatum* LPS or *E. coli* LPS was 4 PM USA Central-Standard-Time (Coordinated Universal Time + 5 h). After the 18 h incubation, conditioned medium (1 mL) from each tissue culture well was split into two aliquots. One aliquot (0.1 mL) was used to measure lactate dehydrogenase (LDH) levels and the remaining aliquot (0.9 mL) was frozen (−84 °C) until determination of TXB_2_, chemokines, and cytokines as described below. Once the conditioned media had been removed, either *S. javanicum*, *S. ocellatum*, or *E. coli* LPS–treated microglia cells were washed with warm (37 °C) HBSS, and O_2_^−^ was determined as described below.

### 5.5. Assay for Microglia O_2_^−^ Generation

O_2_^−^ generation was determined by the SOD-inhibitable reduction of FCC [19]. Briefly, PMA (1 µM)-triggered O_2_^−^ release from either *S. javanicum*, *S. ocellatum* or *E. coli* LPS-activated microglia was measured in the presence of FCC (50 μM) and HBSS, with or without SOD (700 Units), which inhibited >95% of FCC reduction during a 70 min incubation. All experimental treatments were run in duplicate and in a final volume of 1 mL. Changes in FCC absorbance were measured at 550 nm using a DU-800 spectrophotometer (Beckman Coulter, Inc., Indianapolis, Indiana, USA). Differences in the amount of reduced FCC, in the presence and absence of SOD, were used to determine microglia O_2_^−^ generation using the molecular extinction coefficient of 21.0 × 10^3^ M^−1^ cm^−1^ and data expressed in nmol.

### 5.6. Lactate Dehydrogenase Assay

To assess cell viability following preincubation of microglia with either *S. javanicum*, *S. ocellatum* or *E. coli* LPS as described in our experimental protocol, the conditioned medium was harvested and LDH release was determined spectrophotometrically [19,60]. Microglia LDH release was expressed as a percent of total LDH released into the conditioned media. Total LDH release resulted from 0.1% Triton X-100-lysed microglia (intracellular LDH) plus LDH present in the extracellular media, because the fetal bovine serum contained LDH (data not shown). Unless LDH release from LPS-treated microglia was significantly greater than that observed from the vehicle-treated group, shown as 0 or control in the corresponding figures, the 18 h LPS treatment was considered to have had no effect on microglia viability.

### 5.7. Assay for Microglia TXB_2_ Generation

After preincubation of neonatal rat microglia with either *E. coli*, *S. javanicum*, or *S. ocellatum* LPS for 18 h, TXB_2_ production was determined by immunoassay (Cayman Chemical, Ann Arbor, MI, USA) according to the manufacturer’s protocol. Results were expressed as pg/mL and the minimum detectable concentration was 7.8 pg/mL TXB_2_.

### 5.8. Assay for Microglia MMP-9 Generation

After 18 h treatment of neonatal rat microglia with *E. coli*, *S. javanicum*, or *S. ocellatum* LPS, MMP-9 generation was determined by ELISA (Cat# DY8174-05, R&D Systems, Minneapolis, MN, USA) according to manufacturer’s protocol. Results were expressed as pg/mL and the minimum detectable concentration was 78.10 pg/mL MMP-9.

### 5.9. Milliplex MagPix Multiplex Array

Supernatants from untreated, *E. Coli* LPS, *S. javanicum* LPS, and *S. ocellatum* LPS-treated microglia were added to a 96 well Milliplex kit plate (Cat# RECYTMAG-65K, Millipore, Billerica, MA, USA) to assay the following cytokines and chemokines: TNF-α, IL-6, CINC-1/CXCL-1, MIP-1α/CCL3, MIP-2/CXCL-2, and IL-10. The Milliplex plate was read by the Luminex MagPix technology. Data was analyzed using xPONENT software (Luminex, Austin, TX, USA). Results were expressed as pg/mL. Minimum detectable concentrations for cytokines and chemokines were: IL-6, 30.7 pg/mL; IL-10, 2.7 pg/mL; TNF-α, 1.9 pg/mL; CINC-1/CXCL1, 19.7 pg/mL; MIP-2/CXCL2, 11.3 pg/mL; and MIP-1α/CCL3, 0.8 pg/mL.

### 5.10. Statistical Analysis of the Data

Data was expressed as means ± SEM of triplicate determinations of 3 similar experiments. Data was analyzed with Prism software package version 7 from GraphPad (San Diego, CA, USA). Appropriate multiway analysis of variance was performed on all sets of data. Where significant interactions were encountered, simple effects were tested with a one-way analysis of variance followed by a Dunnett multiple comparisons test. Differences were considered statistically significant at *p* < 0.05 [3].

## Supplementary Materials

The following is available online: http://www.mdpi.com/2072-6651/10/4/130/s1. Supplementary Table S1: Effect of *S. javanicum* and *S. ocellatum* LPS on Neonatal Rat Brain Microglia proinflammatory TXB_2_ Generation.
