# Supplementary material for: Cyanobacteria Scytonema javanicum and Scytonema ocellatum Lipopolysaccharides Elicit Release of Superoxide Anion, Matrix-Metalloproteinase-9, Cytokines and Chemokines by Rat Microglia In Vitro
Source: Toxins (Basel). 2018 Mar 21;10(4):130. doi: 10.3390/toxins10040130 (PMC5923296; doi:10.3390/toxins10040130)
Supplement: Supplementary file 1 [file toxins-10-00130-s001.pdf]

# Supplementary Material: Cyanobacteria *Scytonema javanicum* and *Scytonema ocellatum* Lipopolysaccharides Elicit Release of Superoxide Anion, Matrix-Metalloproteinase-9, Cytokines and Chemokines by Rat Microglia In Vitro

**Lucas C. Klemm, Evan Czerwonka, Mary L. Hall, Philip G. Williams and Alejandro M. S. Mayer**

**Table S1.** The effect of *E. coli*, *S. javanicum*, and *S. ocellatum* LPS on neonatal rat microglia $TXB_2$ release.

| $TXB_2$ Release | | | | | | |
|---|---|---|---|---|---|---|
| **LPS** | ***E. coli*** | | ***S. javanicum*** | | ***S. ocellatum*** | |
| [ng/mL] | [pg/mL] | n [a] | [pg/mL] | n [a] | [pg/mL] | n [a] |
| 0 | 6748.2 ± 613.3 | 3 | 6748.2 ± 613.3 | 3 | 6748.2 ± 613.3 | 3 |
| 0.1 | 4683.0 ± 984.4 | 3 | 4649.3 ± 2641.3 | 2 | 4593.8 ± 2188.1 | 2 |
| 1 | 6488.8 ± 1278.4 | 3 | 5947.4 ± 384.7 | 3 | 6812.9 ± 645.4 | 3 |
| 10 | 6795.3 ± 1266.6 | 3 | 6120.7 ± 1,218.9 | 3 | 6553.9 ± 756.6 | 3 |
| 100 | 7698.5 ± 1779.9 | 3 | 6207.3 ± 687.9 | 3 | 6545.1 ± 712.2 | 3 |
| 1,000 | ND | | 6435.5 ± 395.8 | 3 | 7345.9 ± 827.9 | 3 |
| 10,000 | ND | | 6806.5 ± 1320.0 | 3 | 6959.0 ± 1282.0 | 3 |
| 100,000 | ND | | 8547.1 ± 1073.6 | 3 | 7328.5 ± 547.5 | 3 |

[a] Neonatal rat microglia (1.8–2.0 × $10^5$ cells/well) were treated with *E. coli* LPS [0.1–100 ng/mL], *S. javanicum* LPS [0.1–1 × $10^5$ ng/mL], or *S. ocellatum* LPS [0.1–1 × $10^5$ ng/mL] for 18 h in vitro. $TXB_2$ was determined as described in Materials and Methods. Data expressed as pg/mL is the mean ± SEM from 2 or 3 independent experiments (n), each with triplicate determinations. ND: Not done.
